# Growth Inhibition of Head and Neck Squamous Cell Carcinoma Cells by sgRNA Targeting the Cyclin D1 mRNA Based on TRUE Gene Silencing

**DOI:** 10.1371/journal.pone.0114121

**Published:** 2014-12-01

**Authors:** Satoshi Iizuka, Nobuhiko Oridate, Masayuki Nashimoto, Satoshi Fukuda, Masato Tamura

**Affiliations:** 1 Department of Otolaryngology-Head and Neck Surgery, Graduate School of Medicine, Hokkaido University, Sapporo, Japan; 2 Department of Otorhinolaryngology, Head and Neck Surgery, Yokohama City University School of Medicine, Yokohama, Japan; 3 Research Institute for Healthy Living, Niigata University of Pharmacy and Applied Life Sciences, Niigata, Japan; 4 Department of Biochemistry and Molecular Biology, Graduate School of Dental Medicine, Hokkaido University, Sapporo, Japan; Queen Mary University of London, United Kingdom

## Abstract

Head and neck squamous cell carcinoma (HNSCC) exhibits increased expression of cyclin D1 (CCND1). Previous studies have shown a correlation between poor prognosis of HNSCC and cyclin D1 overexpression. tRNase ZL-utilizing efficacious gene silencing (TRUE gene silencing) is one of the RNA-mediated gene expression control technologies that have therapeutic potential. This technology is based on a unique enzymatic property of mammalian tRNase ZL, which is that it can cleave any target RNA at any desired site by recognizing a pre-tRNA-like complex formed between the target RNA and an artificial small guide RNA (sgRNA). In this study, we designed several sgRNAs targeting human cyclin D1 mRNA to examine growth inhibition of HNSCC cells. Transfection of certain sgRNAs decreased levels of cyclin D1 mRNA and protein in HSC-2 and HSC-3 cells, and also inhibited their proliferation. The combination of these sgRNAs and cisplatin showed more than additive inhibition of cancer cell growth. These findings demonstrate that TRUE gene silencing of cyclin D1 leads to inhibition of the growth of HNSCC cells and suggest that these sgRNAs alone or combined with cisplatin may be a useful new therapy for HNSCCs.

## Introduction

Head and neck squamous cell carcinoma (HNSCC) is a common malignancy and accounts for 550,000 new cases worldwide every year [1]. Patients with HNSCC are treated by a combination of surgery, radiation therapy and chemotherapy. Despite recent advances in therapy including novel cytotoxic chemotherapeutic agents, which have improved quality of life, survival rates have remained static for many years [1], [2] . Therefore, it is essential that we develop more effective therapies.

The most critical point in regulation of the cell cycle is the G1 check-point. Cyclin D1, a G1 cyclin, has been implicated in regulation of the G1 to S phase progression in many different cell types. Together with its binding partners cyclin-dependent kinase (CDK) 4 and CDK6, cyclin D1 forms active complexes that promote the phosphorylation of retinoblastoma protein (RB) and activation of E2F-responsive gene with roles in DNA synthesis, and in turn promote progression through the G1 phase of the cell cycle [3], [4]. CCND1 (a gene of cyclin D1) is a well-established human oncogene. Human CCND1 is located on chromosome 11q13 where DNA rearrangement and amplification have been detected in several types of human cancers including HNSCC [5], [6]. Overexpression of cyclin D1 is much more common than can be accounted for by copy number or by mutations that affect CCND1 expression. Cyclin D1 mRNA and protein overexpression is a consequence of oncogenic activation of several mitogenic signaling pathways (such as the Ras-MEK-ERK and PI3K pathways). Many common cancers have CCND1 amplification rates of 15–40%, and higher rates of cyclin D1 mRNA and protein overexpression [4]. Some studies have reported that cyclin D1 is overexpressed in 19% to 68% of HNSCCs [7], [8].

Data from several clinical studies indicate that cyclin D1 expression is a biomarker of cancer phenotype and disease progression in several cancers. Multiple studies have found a significant association between high levels of cyclin D1 expression and shorter patient survival in many cancers and high expression of cyclin D1 is often associated with increased metastasis [9]–[11]. In tumors from HNSCC patients, those with cyclin D1-positive tumors had a poor prognosis associated with lymph node metastasis, recurrence and shorter patient survival compared with cyclin D1-negative tumors, indicating a potential use for these markers in predicting the clinical outcome of HNSCC patients [12], [13]. Consequently, cyclin D1 is an attractive therapeutic target. However, cyclin D1 is regarded as difficult to target directly. Instead, several small molecular CDK inhibitors that block the associated kinase are undergoing clinical testing [4], [14]. To date, these CDK inhibitors have had limited success. Another approach, using mTOR inhibitors that block the translation of cyclin D1 mRNA, show potential but are less well developed [15]. Therefore, studies are still required to elucidate effective knock-down methods directed towards cyclin D1 itself for use as cancer therapy.

tRNase ZL-utilizing efficacious gene silencing (TRUE gene silencing) is one of the RNA-mediated gene expression control technologies that have therapeutic potential [16]–[21]. This method is based on a unique enzymatic property of mammalian tRNase ZL, which is that it can cleave any target RNA at any desired site by recognizing a pre-tRNA-like or micro-pre-tRNA-like complex formed between the target RNA and an artificial small guide RNA (sgRNA). We have demonstrated the efficacy of TRUE gene silencing by using it to introduce into living cells various artificially-designed sgRNAs either as their expression plasmids or as 2′-O-methyl RNAs [18], [22]. sgRNA is divided into four types, 5′-half-tRNA, RNA heptamer, hook RNA, and 14-nt linear RNA [16], [18], [23], [24]. The efficacy of TRUE gene silencing can be close to that of RNA interference technology [21]–[23]. sgRNA can be easily taken up by cultured cells without any transfection reagents, and naked sgRNAs targeting Bcl2 or WT1 mRNA can reduce the mRNA level and the amount of protein as well as inducing apoptosis of leukemia cells [24], [25]. sgRNAs have advantages in that they are easier, more accurate and cheaper to synthesize than longer RNAs and that cells appear to take them up more easily [24]–[26].

In this study, we designed several sgRNAs targeting human cyclin D1 mRNA and examined the effects on squamous cell carcinoma (SCC) cells. Transfection of certain effective sgRNAs decreased levels of cyclin D1 mRNA and protein in HSC-2 and HSC-3 cells, and they also prohibited cell cycle progression and cell growth. The combination of these sgRNAs with cisplatin showed more than additive inhibition of cancer cell proliferation. These findings demonstrate that TRUE gene silencing targeted to cyclin D1 leads to inhibition of proliferation of SCC cells and suggest that these sgRNAs may have potential to be therapeutically useful for several cancers including HNSCC.

## Materials and Methods

### RNA synthesis and preparation

The 5′- and 3′-phosphorylated sgRNAs (sgHT1-6, sgL2, 5 and sgH2, 5) with full 2′-O-methyl modifications were chemically synthesized using a DNA/RNA synthesizer and subsequently purified by high-performance liquid chromatography with a buffer containing acetonitrile/triethylammonium acetate by Nippon Bioservice (Asaka, Saitama, Japan). Alexa568-3′-labeled sgRNAs were also chemically synthesized. Nucleotide sequences of sgRNAs are shown in Table 1. Silencer select pre-designed small interfering RNA (siRNA) for mouse cyclin D1 (Ambion, ID # s229) was used as the positive control. Unrelated heptamer RNA, sgLucHep1; 5′-GGGCCAG-3′, sgLucHep2; 5′-GAUCGAG-3′, sgLucHep3; 5′-GAGCGAG-3′, H5470; 5′-pUUUUUCUp-3′ and H13782; 5′-pCUUCUUUp-3′ were used as negative controls [18], [26].

**Table1:** Sequence of sgRNAs.

Name	Sequence(5′ to 3′)
HT1	5′-pGCAGCAGUGGCGCAAUGGAUAACGCGUUGUC-3′
HT2	5′-pCCCGCUGUGGCGCAAUGGAUAACGCGAUUGG-3′
HT3	5′-pGAUGUGCUGGCGCAAUAACGCGUGUCA-3′
HT4	5′-pGGCACCGUGGCGCAAUGGAUAACGCGGCCUC-3′
HT5	5′-pCGGCCUCUGGCGCAAUGGAUAACGCGCGCCA-3′
HT6	5′-pACACUUGUGGCGCAAUGGAUAACGCGUAAUA-3′
H2	5′-pCCCGCUG-3′
L2	5′-pCCCGCUGCCACCAU-3′
H5	5′-pCGGCCUC-3′

### Reagents

Cisplatin (cis-diammine dichloroplatinum; CDDP) was obtained from Yakuruto Corp. (Tokyo, Japan).

### Cell culture and transfection

Human squamous cell carcinoma (SCC) cells, of the HSC-2 or HSC-3 cell line (obtained from RIKEN BioResource Center, Tsukuba, Japan) [27] were cultured in RPMI-1640 medium (Sigma-Aldrich, St. Louis, MO) containing 100 µg/mL kanamycin (Meiji, Tokyo, Japan) and 10% fetal bovine serum (FBS; PAA Laboratories; Pasching, Austria) at 37°C in cell culture dishes (Corning, Corning, NY) in a humidified atmosphere of 5% CO_2_. Cells of the human embryonic kidney 293 line (HEK293), were cultured in Dulbecco's modified Eagle's medium (DMEM, Sigma-Aldrich) containing 100 µg/mL kanamycin supplemented with 10% FBS. Human cervical carcinoma Hela cells [28], human osteosarcoma MG-63 cells [29] (obtained from RIKEN BioResource Center, Tsukuba, Japan) and primary human gingival fibroblasts (ScienCell Research Labortories, Carlsbad, CA) were cultured in alpha-minimal essential medium (α-MEM, Sigma-Aldrich) containing 100 µg/mL kanamycin supplemented with 10% FBS. Cells were transfected with sgRNA that specifically targets human cyclin D1, or with siRNA using Lipofectamine 2000 (Invitrogen, Carlsbad, CA) according to the manufacturer's protocol, or with no transfection reagent.

### RNA extraction and quantitation of gene expression by reverse transcription-polymerase chain reaction (qRT-PCR)

Total RNA was extracted from the cells at the indicated time-points using Isogen II (Nippongene, Toyama, Japan). Complementary DNA was synthesized using high capacity cDNA reverse transcription kits (Applied Biosystems, Foster City, CA) according to the manufacturer's instructions. Quantitative RT-PCR (qRT-PCR) was performed using assay-on-demand TaqMan probes (Hs00765553_m1, Applied Biosystems) and the ABI Prism 7000 sequence detection system as previously described [30]. The relative level of gene expression was quantified using the comparative Ct method with glyceraldehyde-3-phosphate dehydrogenase (GAPDH) as the endogenous control.

### Western blot analysis

Cells were washed with ice-cold PBS and suspended in CelLytic-M Mammalian cell lysis/extraction reagent (Sigma) plus a protease inhibitor (Complete mini, Roche, Indianapolis, IN) and phosphatase inhibitor cocktail PhoSTOP (Roche, Basel, Switzerland). Whole cell extracts were separated by 4–15% gradient SDS polyacrylamide Mini-PROTEAN TGX gel electrophoresis (Bio-Rad Laboratories, Richmond, CA), and transferred to a PVDF membrane (Millipore, Bedford, MA). The membrane was probed with polyclonal antibodies raised to anti-cyclin D1 (Sc8396, Santa Cruz Biotechnology, Santa Cruz, CA), anti-phosphoRB (#8516, Ser807/811, Cell Signaling Technology, Danvers, MA), anti-RB (#9313, Cell Signaling) or anti-β-actin antibodies (GeneTex, Irvine, CA) using the ECL prime detection system (GE lifesciences, Pittsburgh, PA) according to the manufacturer's instructions.

### Localization of sgRNA in living cells

To visualize intracellular sgRNA, HSC-3 cells (1×10^5^ cells/well) were seeded into collagen-coated glass-bottomed dishes (Matsunami glass Inc., Osaka, Japan). After 24 h, the cells were treated with 200 nM naked Alexa568-3′-labeled sgRNA, and then cultured for a further 24 h. The cells were then rinsed twice with 1× phosphate-buffered saline (PBS), and then an inverted microscope (Nikon, Ti-E, Tokyo, Japan) equipped with a Plan Fluor 40x objective lens (NA 0.75, Nikon) or a Plan Apo VC 100x objective lens (NA 1.40, Nikon) and micro scanning stage (BI XY stage, Chuo Precision Industrial Co. Ltd., Tokyo, Japan) was used to observe fluorescence images in living cells maintained at 37°C with a continuous supply of 95% air and 5% CO_2_ using a stage-top incubator (INUBG2TF-WSKM, Tokai Hit, Fujinomiya, Japan). The nuclei or mitochondria were visualized with Hoechst 33342 (H21492, Molecular Probes, Invitrogen, Eugene, OR) or MitoTracker Green FM (Molecular Probes), respectively. The fluorescent cells were counted randomly at least 15 fields under the fluorescence microscope with a 40× objective lens and calculated as a percentage of the total number of fluorescent cells.

### Analysis of cell cycle progression using the fluorescence ubiquitination cell cycle indicator (FUCCI)

To investigate cell cycle progression and division in live cells, we used the fluorescent ubiquitination-based cell cycle indicator (FUCCI) which uses E3 ligase substrates, Cdt1 and geminin, fused to red fluorescent protein (Cdt1-RFP) and green fluorescent protein (geminin-GFP), to track the G1/G0 phase and S/G2/M phases, respectively, during cell cycle progression [31]. HSC-3 cells (1×10^4^ cells/well) were seeded into collagen-coated 35-mm glass-bottomed dishes (Matsunami glass Inc.) and cultured for 24 h. At the end of this period the cells were treated with 200 nM naked sgRNA, cultured for an additional 24 h, then transduced with geminin-GFP and Cdt1-RFP constructs, packaged in the BacMam gene delivery system (Premo FUCCI Cell Cycle Sensor BacMam 2.0, Life Technologies, Carlsbad, CA) according to the manufacturer's protocol. In brief, transduction solution was prepared by adding 40 µL of Premo geminin-GFP and 40 µL of Permo Cdt1-RFP into 2 mL of culture medium. Cell culture medium was replaced with 2 mL of transduction solution and the cells were incubated at 37°C for 1 h, and then incubated at room temperature for another 1 h. Following the incubation, the transduction solution was removed and 1× BacMam enhancer (Life Technologies) in medium was added to the cells and incubated for 90 min at 37°C. The solution was then removed, medium was added to the cells and they were grown for another 16 h. Confocal images were captured using a confocal laser scanning microscopy system (Nikon A1 and Ti-E, Nikon) equipped with a Plan Apo VC 20x objective lens (NA 0.75, Nikon) and a stage-top incubator (INUBG2H-TIZB, Tokai Hit). The red and green fluorescent cells were counted under the fluorescence microscope and calculated as a percentage of the total number of fluorescent cells.

### Measurement of caspase 3/7 activity

The cells were labeled with 2 µM CellEvent Caspase-3/7 green detection reagent (Life Technologies) which is a nucleic acid-binding dye that harbors the caspase-3/7 cleavage sequence, DEVD, and fluoresces after being cleaved and bound to DNA. After incubation for 30 min at 37°C in a humidified atmosphere of 5% CO_2_, fluorescence images were observed by an inverted microscope (Nikon, Ti-E) equipped with a Plan Fluor 40x objective lens (NA 0.75, Nikon) maintained at 37°C with a continuous supply of 95% air and 5% CO_2_ using a stage-top incubator (INUBG2TF-WSKM, Tokai Hit). Images were captured using a cooled CCD camera (ORCA-R2, Hamamatsu Photonics).

Cellular enzymatic activities of caspases 3/7 were determined by a caspase colorimetric assay (Caspase-Glo 3/7 Assay Systems, Promega, Madison, MI) according to the manufacturer's instructions. Briefly, for each reaction, cells were lysed and incubated with a luminogenic substrate containing the DEVD sequence, which is cleaved by activated caspase 3/7. After incubation at room temperature for 3 h, luminescence was quantified with a luminometer (Glomax 20/20, Promega).

### Detection of DNA synthesis by chemiluminescent bromodeoxyuridine (BrdU) ELISA

To measure cell proliferation, newly synthesized DNA of replicating cells was assayed by BrdU incorporation using a BrdU labeling and detection ELISA-kit (Cell Proliferation Biotrak ELISA System version 2, GE Healthcare) according to the manufacturer's instructions. Briefly, BrdU was added to the cells. After 24 h, cells were fixed and DNA denatured, then incubated with an antibody to BrdU conjugated with peroxidase (60 min, 37°C). Immune complexes were detected by incubation with tetramethylbenzidine as substrate for 5 min, the reaction was stopped with H_2_SO_4_ and absorption measured at 450 nm in a microplate reader (iMark, Bio-Rad).

### Quantitation of living cell numbers

To quantitate cell viability, the tetrazolium-based colorimetric CCK-8 assay (Dojindo Laboratories, Kumamoto, Japan) was used. A 20 µL aliquot of the substrate WST-8 (2-(2-methoxy-4-nitrophenyl)-3-(4-nitrophenyl)-5-(2,4-disulfophenyl)-2H-tetrazolium, monosodium salt) was added to each well. After incubation for 2 h at 37°C, the optical density was measured at a wavelength of 450 nm using a microplate reader (Varioskan Flash 2.4, Thermo Fisher Scientific, Waltham, MA).

### Statistical analysis

All experiments were repeated at least three times and representative results are shown. In the qRT-PCR analysis, BrdU incorporation and cell viability assay, differences between control and experimental groups are reported as the mean ± standard deviation (SD), and were analyzed by Student's t-test, in which values of *P*<0.05 were considered significant.

## Results

### Design of sgRNAs targeting human cyclin D1 mRNA and reduction in cyclin D1 mRNA and protein levels by sgRNAs

**Figure 1:**
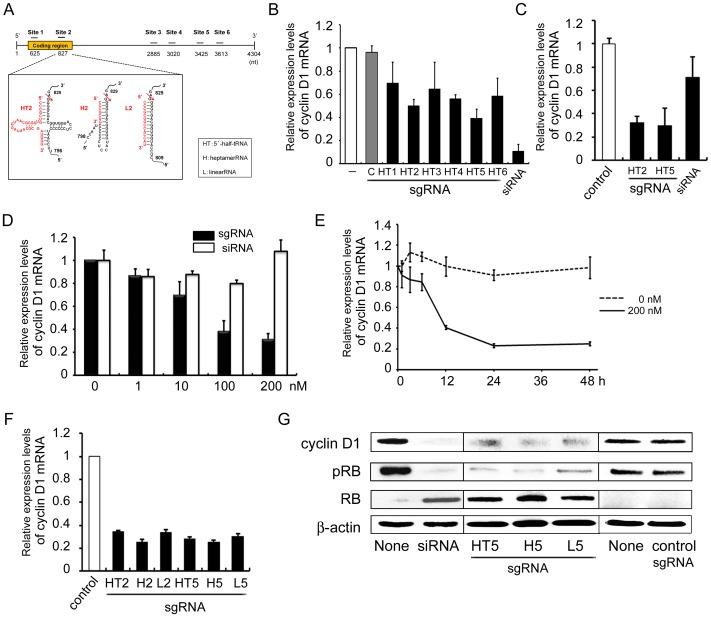
Design of an sgRNA targeting human cyclin D1 mRNA and the reduction in cyclin D1 mRNA and protein level by sgRNA. (A) Design of an sgRNA targeting human cyclin D1 mRNA. Secondary structures of sgRNA/target complexes between the sgRNA and the human cyclin D1 mRNA. Arrows indicate the expected tRNase ZL cleavage sites, with numbers at the cleavage sites from GenBank data (#NM_053056.2). (B and C) Reduction in cyclin D1 mRNA level following sgRNA transfection. HSC-2 cells were plated at 1×10^5^ cells/cm^2^, and then cultured for 24 h. (B) sgHT1–6 (200 nM), siRNA (10 nM), sgLucHep3 (C) (10 nM) or vehicle (-) was transfected with transfection reagent; (C) sgHT2 or sgHT5 (200 nM each), siRNA (10 nM) or sgLucHep3 (control) was treated without transfection reagent after which the cells were cultured for a further 24 h. Total RNA was extracted from the cells and the cyclin D1 mRNA level was determined by qRT-PCR. (D) Dose-dependent regulation of cyclin D1 mRNA expression. HSC-3 cells were plated and cultured as above. After 24 h, naked sgHT2 or siRNA was added at the indicated concentration. (E) Time-dependent regulation of cyclin D1 mRNA expression. HSC-3 cells were plated and cultured as above. After 24 h, naked sgHT2 was added at 0 (DASHED LINE) OR 200 nM (SOLID LINE) (E), and the cells were cultured for the indicated period. Cyclin D1 mRNA level was determined by qRT-PCR. (F) Effect of sgRNA subtype on the reduction of cyclin D1 mRNA expression. HSC-3 cells were plated and cultured as above. After 24 h, naked sgHT2, sgH2, sgL2, sgHT5, sgH5, sgL5 or sgLucHep2 (control) was added at 200 nM, after which the cells were cultured for 24 h. Cyclin D1 mRNA level was determined by qRT-PCR. (G) Regulation of the cyclin D1, RB and pRB protein level by sgRNA using western blot analyses. HSC-3 cells were plated and cultured as above. After 24 h, naked sgHT2, sgHT5, sgL5, sgLucHep2 (control) (200 nM each) or siRNA (20 nM) was added using no transfection reagent (sgRNA) or with Lipofectamine 2000 (siRNA), after which the cells were cultured for 24 h. The levels of cyclin D1, RB and pRB protein in the cells were determined by western blot analysis using appropriate antibodies. Blots were re-probed for β-actin as control. Each assay represents a separate experiment performed in triplicate. Data are presented as means ± S.D.

To estimate the silencing effect of the TRUE gene silencing method on cyclin D1 expression in SCC cells, three types of sgRNA: 5′-half-tRNA (HT) type, 14-nt linear RNA (L) type and heptamer RNA (H) type were designed at each of six arbitrary sites of human cyclin D1 mRNA (Genbank accession #NM_053056.2) (Figure 1A). Each HT-type sgRNA can form a pre-tRNA-like structure with the target cyclin D1 mRNA through 7 and 5 base-pairings corresponding to the acceptor and anticodon stems, respectively [16]. The complexes of each H type sgRNA can form co-axially stacked perfect 12-bp stem-loops. When they form tRNA acceptor-stem-like duplexes with target mRNA through base-pairing, the H type sgRNAs can direct efficient specific cleavages of human cyclin D1 RNAs by tRNase ZL [18]. Each L type sgRNA is a 14-nt sequence complementary to a sequence 5′ to the desired cleavage site, and can form a 14-bp double-stranded RNA with the human cyclin D1 mRNA, which roughly corresponds to a combination of the acceptor and T stems [20]. A siRNA which has been shown to work very efficiently was used as a positive control, and an unrelated RNA was used as a negative control.

First, we examined which site of human cyclin D1 mRNA among our designed sites was effective for TRUE silencing of cyclin D1 expression. HSC-2 cells derived from human SCC were transfected with 200 nM of HT type sgRNA (sgHT1-6) or siRNA for cyclin D1 using a transfection reagent (Lipofectamine 2000), and then the mRNA expression level of the cyclin D1 was determined by qRT-PCR (Figure 1B). siRNA for cyclin D1 and control sgRNA (sgLucHep3) were used as positive and negative controls, respectively. Although vehicle or control sgRNA (sgLucHep3) could not decline cyclin D1 mRNA, sgRNAs of sgHT2, sgHT4 and sgHT5 down-regulated cyclin D1 mRNA (Figure 1B). The levels of suppression caused by sgHT2 and sgHT5 were approximately 50 and 39%, respectively, while siRNA caused a reduction of 89%, suggesting that at least two of the designed sites that contain a potential tRNase ZL target site are effective in silencing cyclin D1 expression.

Previously, we have reported that sgRNA can be easily taken up by cultured cells without any transfection reagents, and naked sgRNAs targeting Bcl2 or WT1 mRNA can reduce their mRNA level and the amount of protein as well as inducing apoptosis in leukemia cells [24], [25]. Therefore, sgRNAs were treated without any transfection reagents, and we found that sgHT2 and sgHT5 could down-regulate the mRNA levels to approximately 31 and 29% respectively in HSC-2 cells (Figure 1C), indicating that these sgRNAs targeting cyclin D1 mRNA were just as effective whether or not they were transfected using a transfection reagent.

Next, we examined the dose and time-dependent effects of these effective sgRNAs on inhibition of cyclin D1 expression without transfection reagent. The level of cyclin D1 mRNA decreased by approximately 60% with 100 nM naked sgHT2 and a small further decrease was observed with 200 nM, indicating that sgHT2 reduces target mRNA level dose-dependently in HSC-3 cells (Figure 1D). siRNA treatment without transfection reagent had little effect on the inhibition of cyclin D1 mRNA expression as compared with the same amount of sgRNA. In time-course experiments, treatment with naked sgHT2 decreased cyclin D1 mRNA level to 40% at 12 h and to 23–25% at 24 and 48 h (Figure 1E). To elucidate which type of sgRNA is more effective in silencing cyclin D1 expression, three types of sgRNA were treated into HSC-3 cells. As shown in Figure 1F, any of the types of sgRNA was able to down-regulate cyclin D1 mRNA expression by targeting sites 2 and 5 of cyclin D1 mRNA.

The effects of cyclin D1 protein level following effective sgRNA treatment were evaluated by western blot analysis. Cyclin D1 protein was also diminished by naked sgHT5 as compared with control in HSC-3 cells (Figure 1G). As expected, cyclin D1 protein was decreased by siRNA with transfection reagent (Figure 1G). It is well known that tumor suppressor protein RB represents the critical target of cyclin D1 in cell cycle control. Cyclin D1 initiates the phosphorylation of RB that results in disruption of RB-mediated cell growth repression. Therefore, we estimated the phosphorylation status of RB using an antibody specific to phospho-RB. As expected, sgHT5 down-regulated phosphorylated RB (pRB) level and increased RB protein in HSC-3 cells, suggesting that downregulation of pRB occurs in parallel to the induction of G1 arrest (vide infra) of the tumor cell cycle (Figure 1G). These data confirm that sgRNA targeting cyclin D1 acts to down-regulate not only cyclin D1 itself but also the critical target of cyclin D1 in cell cycle control in tumor cells.

### Confocal microscopic analysis for uptake and intracellular localization of sgRNA without transfection reagents

**Figure 2:**
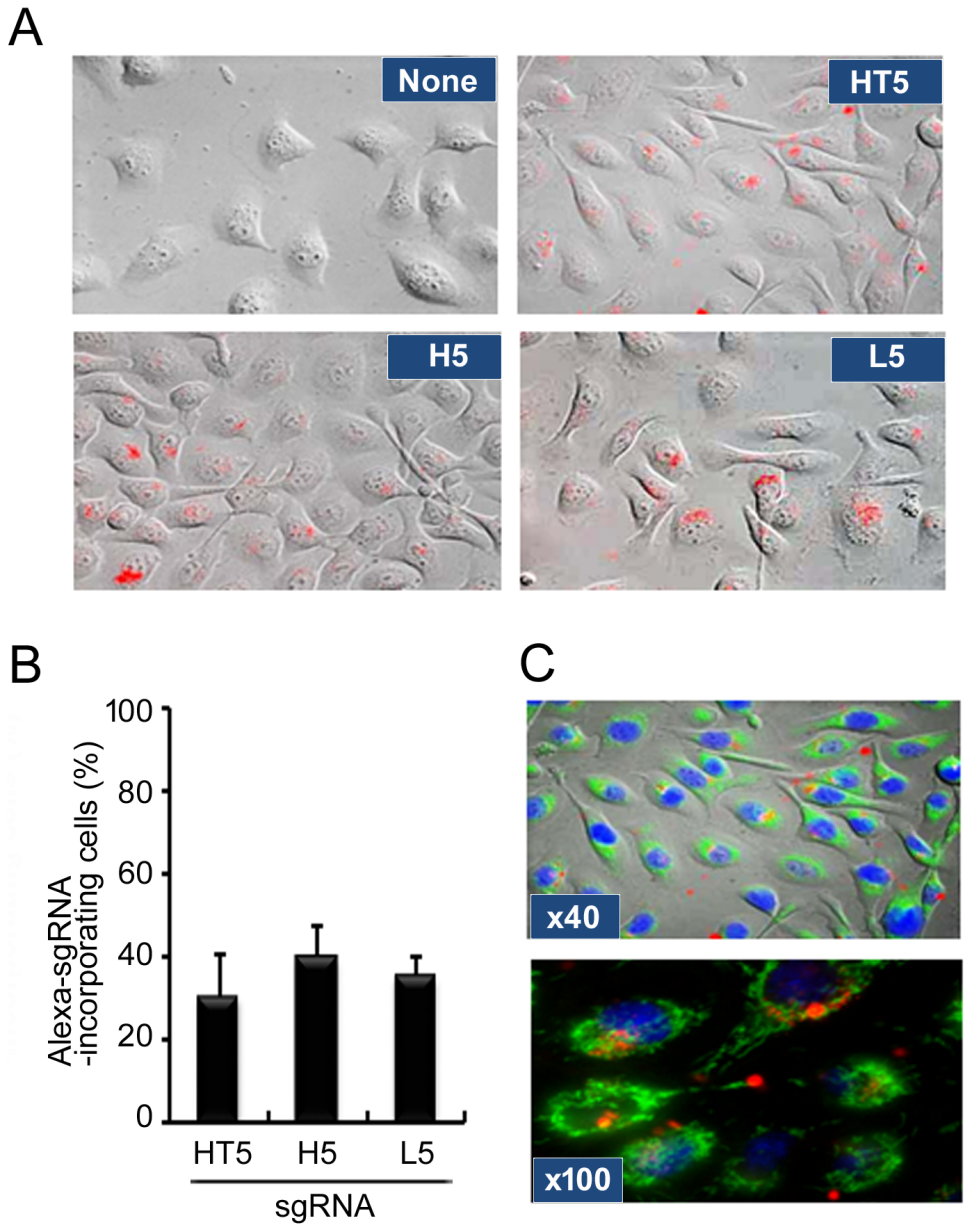
Confocal microscopic analysis for uptake and intracellular localization of sgRNA. (A) HSC-3 cells were plated and cultured for 24 h. Naked Alexa568-3′-labeled sgHT2, sgH5 or sgL5 was added at 200 nM and then cells were cultured for a further 24 h, after which the cells were observed by confocal microscopy as described in Materials and Methods. (B) Red fluorescent cells were counted under a fluorescence microscope and the percentage of stained cells was calculated. Each assay represents a separate experiment performed in triplicate. Data are presented as means ± S.D. (C) HSC-3 cells were treated with naked Alexa568-3′-labeled sgHT5 and then labeled with Mitotracker Green and Hoechst33342 to visualize mitochondria and nuclei, respectively. Cells were observed through a 40x objective lens (upper panel) or 100x objective lens (lower panel) of the microscope.

To directly demonstrate the cellular uptake and intracellular distribution of sgRNA without transfection reagents, we performed fluorescent microscopic imaging analysis using Alexa568-3′-conjugated sgRNA. Confocal microscopy demonstrated that sgHT5, sgH5 and sgL5 were indeed efficiently taken up without any transfection reagents by HSC-3 cells (Figure 2A). Ratios of cellular uptake were very similar (30–40%) between all three types of sgRNA (Figure 2B). Although individual mitochondria or nuclei stained with MitoTracker Green or Hoechst33342 could clearly be seen, a distinct distribution of sgRNA was apparent (Figure 2C). A line scan analysis of fluorescence through sgRNA-Alexa568, Hoechst33432 and Mito Tracker Green also showed distinct differences in distribution between sgRNA and mitochondria (data not shown). The results of 3D analysis of imaging and time-lapse imaging revealed that a large accumulation of sgRNA-Alexa568 was taken up in HSC-3 cells at 6 h after treatment of sgRNA and located in the perinuclear compartment (Video S1, S2 and Methods S1). These results indicated that sgRNA can be taken up by SCC cells without any transfection reagents and located in the cytoplasmic compartment.

### The effects of sgRNA targeting cyclin D1 on the cell cycle and apoptosis

**Figure 3:**
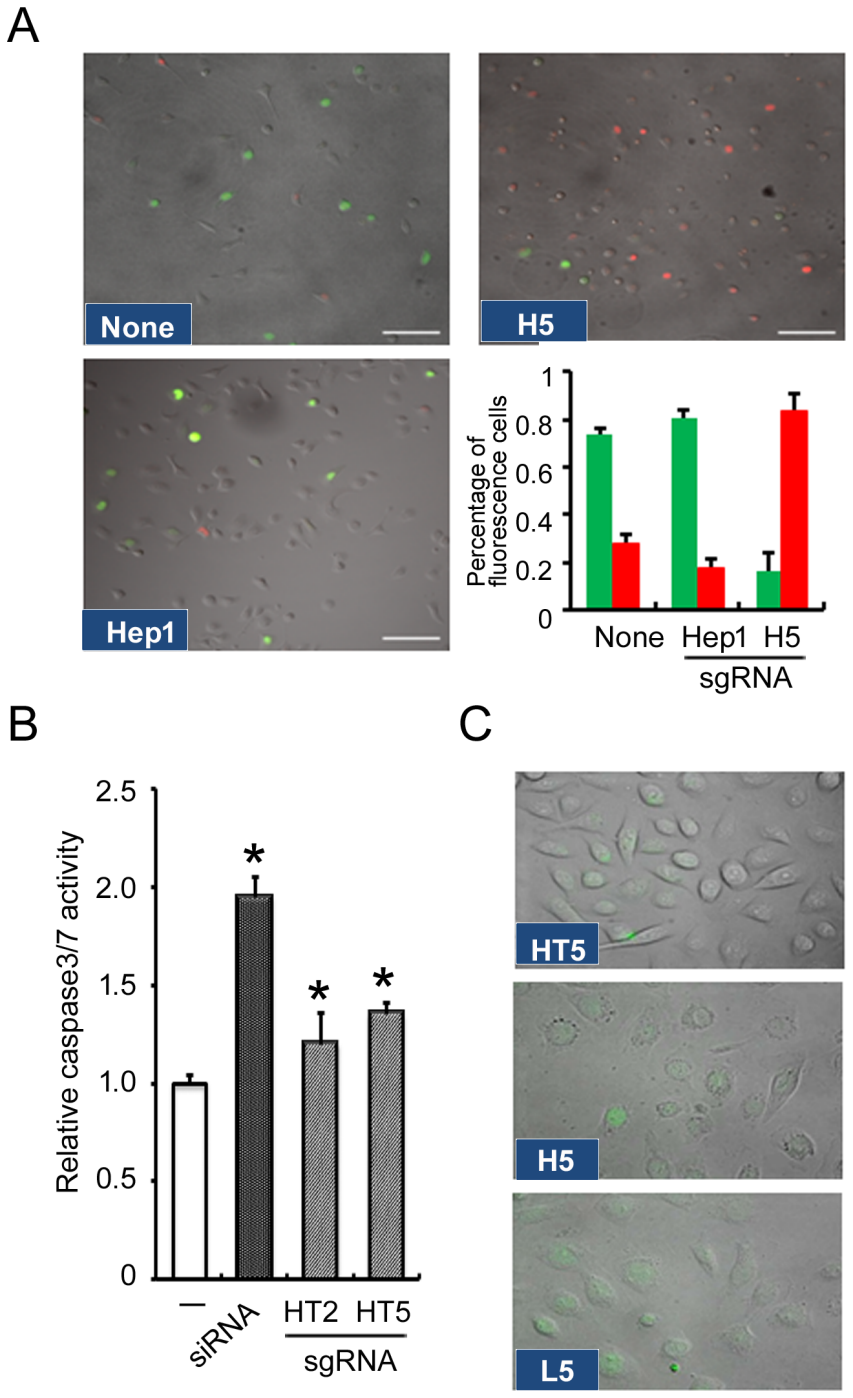
Effects of sgRNA targeting cyclin D1 on cell-cycle and apoptosis. (A) Real-time visualization of cell cycle progression using a fluorescence microscope. HSC-3 cells were plated and cultured for 24 h. *In vitro* visualization of cell cycle distribution in a fluorescent ubiquitination-based cell cycle indicator (FUCCI) was used. Naked sgH5, sgLucHep1 (unrelated heptamer) or vehicle (None) was added at 200 nM and then cell cycle distribution was monitored using a fluorescence microscope. Scale bars  = 100 µm. (B) Cellular activities of caspases 3/7 were measured 6 h after HSC-3 cells were cultured in the absence (–) or presence of naked sgRNA (200 nM each). siRNA (20 nM) for cyclinD1 with Lipofectamine 2000 was used as positive control. Fold-increase in activity was calculated based on activity measured from control cells. (C) Caspase 3/7 activity of cells treated with sgRNA targeting cyclin D1. HSC-3 cells were plated and cultured for 24 h. Naked sgHT5, sgH5 or sgL5 was added at 200 nM and then caspase 3/7 activity was visualized by a caspase 3/7 Green Detection Reagent. Each assay represents a separate experiment performed in triplicate. Data are means ± S.D.*P<0.05.

To better understand the effects of sgRNA targeting of cyclin D1 on cell cycle regulation, we examined cell cycle progression *in vitro* after exposure to sgRNA using the FUCCI system, which normally enables G1 and S/G2/M cells to emit red and green fluorescence, respectively. HSC-3 cells, either untransfected or transfected with control sgRNA, exhibited a combination of red and green nuclei and proliferated in parallel to cell cycle progression. Upon treatment of HSC-3 cells with sgH5, the number of cells with red nuclei gradually increased and those with green nuclei disappeared within 24 h of exposure to sgRNA (Figure 3A), along with reductions of both cyclin D1 mRNA and protein level (Figure 1). These observations suggested that G1 arrest was induced by sgRNA targeting of cyclin D1.

However, the molecular mechanisms connecting cell-cycle arrest and apoptosis are not well understood. We examined caspase 3/7 activity which is activated with apoptosis induction by the sgRNA targeting cyclin D1. Caspase 3/7 activities were elevated to similar levels after exposure to sgHT2, sgHT5 and siRNA targeting cyclinD1 in HSC-3 cells (Figure 3B). From microscopic observations, activated caspase-3/7 fluorescent signals were also detected following treatment with sgRNA (sgHT5, sgH5, sgL5) in HSC-3 cells (Figure 3C), indicating that induction of apoptosis might be due to cell cycle arrest in these cells caused by sgRNA targeting of cyclin D1. From time-lapse imaging analysis, it was apparent that apoptosis was induced in a subset of the HSC-3 cells which had taken up sgH5-Alexa568 (Video S2 and Methods S1).

### The effects of sgRNA targeting of cyclin D1 and cisplatin on living cell numbers and cell proliferation of SCC cells

**Figure 4:**
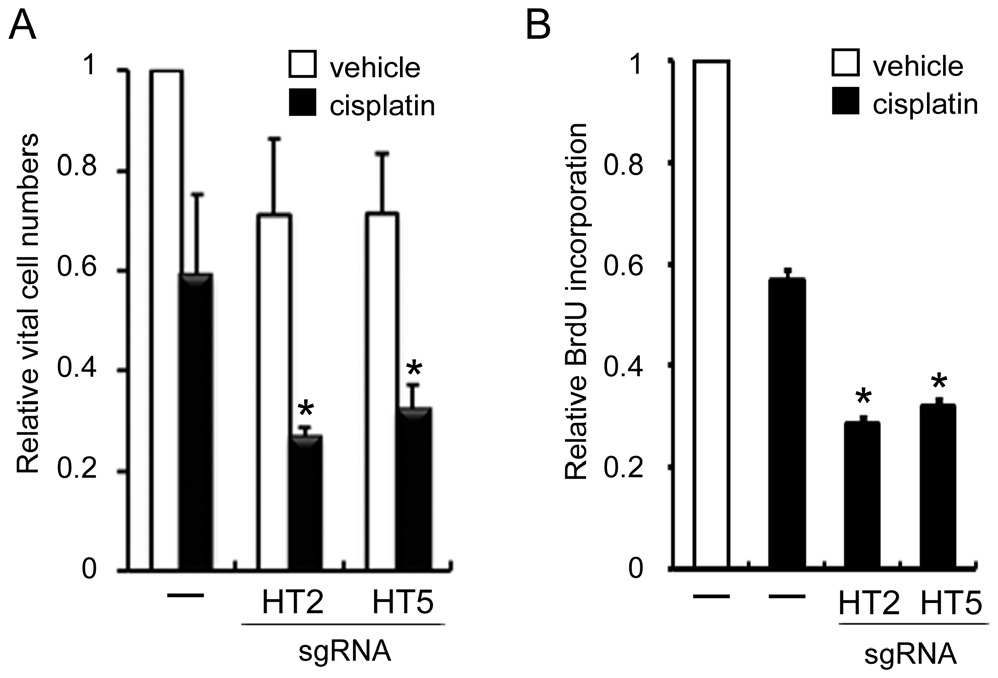
Effects of sgRNA targeting cyclin D1 on viable cell numbers and cell proliferation. HSC-3 cells were plated at 1×10^5^ cells/well in 24 well dishes, and then cultured for 24 h. sgHT2 or sgH5 was added at 200 nM and then cultured for a further 24 h. Cisplatin (10 µM) or vehicle was added to cells, after which the cells were cultured for 72 h. The living cell numbers were quantitated as described in Materials and Methods (A). Cell proliferation was measured by BrdU incorporation using an ELISA kit (B). The relative living cell numbers and BrdU incorporation in the absence of both sgRNAs and cisplatin are adjusted to 1. Each assay represents a separate experiment performed in triplicate. Data are means ± S.D. *P<0.05.

Next, we examined how treatment of sgRNA targeting cyclin D1 affects tumor cell numbers. When sgHT2 or sgHT5 was added to the culture medium without any transfection reagents, living cell numbers of both cell types decreased to approximately 70% in HSC-3 cells (Figure 4A; unfilled bars). These observations suggested that the reduction in HSC cell numbers might be due to both cell cycle arrest and induction of apoptosis by sgRNA targeting of cyclin D1.

Cisplatin is active against several solid malignancies, including HNSCC. Therefore, to examine the effects of the combination of sgRNA targeting cyclin D1 and cisplatin on SCC proliferation, we performed a tumor cell viability assay. The combination of sgHT2 or sgHT5 and cisplatin showed more than additive inhibition of cell number in HSC-3 cells (Figure 4A; solid bars). Furthermore, inhibition of cell proliferation was confirmed by 5-bromo-2′-deoxyuridine (BrdU)-incorporation, which correlates with DNA-synthesis in S phase of the cell cycle. After addition of both cisplatin and naked sgHT2 or sgHT5, the uptake of BrdU by HSC-3 cells reduced additively compared with cisplatin alone (Figure 4B). These findings demonstrate that the combination of sgRNA targeting cyclin D1 with cisplatin leads to inhibition of the growth of SCC cells and indicated that these sgRNAs combined with cisplatin may be useful for therapy of HNSCCs.

### The effects of sgRNA targeting of cyclin D1 on cyclin D1 expression and cell proliferation in other human cancer cell lines and normal cells

**Figure 5:**
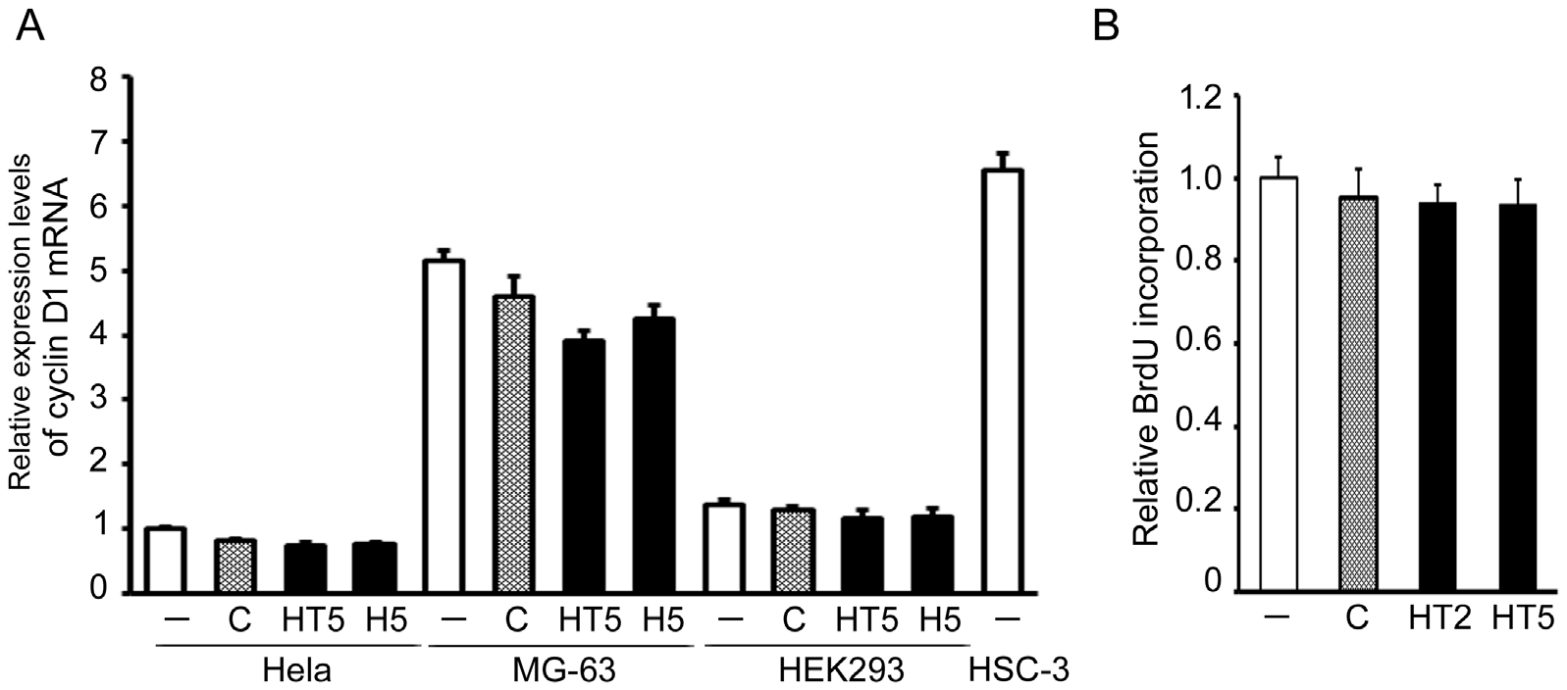
The effects of sgRNA targeting of cyclin D1 on cyclin D1 expression and cell proliferation of other human cancer cell lines and normal cells. (A) Hela, MG-63, HEK293 and HSC-3 cells were plated at 1×10^5^ cells/well in 24 well dishes, and cultured for 24 h. sgHT5, sgH5, H5470 (control, C) or vehicle (-) was added at 200 nM and then the cells cultured for another 24 h. Cyclin D1 mRNA level was determined by qRT-PCR. (B) Cell proliferation of HEK293 cells was measured by BrdU incorporation using an ELISA kit. The cyclin D1 mRNA level and BrdU incorporation in the absence of sgRNAs are adjusted to 1. Each assay represents a separate experiment performed in triplicate. Data are means ± S.D.

In order to elucidate the effects of sgRNA targeting of cyclin D1 on other human cancer cells, we examined cyclin D1 expression in cervical carcinoma Hela cells and osteosarcoma MG-63 cells following the addition of naked sgHT5 and sgH5, using qRT-PCR. These effective sgRNAs could decline the cyclinD1 mRNA expression in MG-63 cells but not Hela cells (Figure 5A). Although confocal microscopy demonstrated that sgH5 was indeed taken up without any transfection reagents by normal fibroblasts (Figure S1), we found that these effective sgRNAs hardly affect the cyclinD1 mRNA expression level or viability of normal human kidney epithelial HEK293 cells (Figures 5A and 5B). Endogenous cyclin D1 mRNA expression level without treatment was higher in HSC-3 cells and MG-63 cells than in other cancer cells (Hela cells) and HEK293 cells (Figure 5A), suggesting that efficiency of sgRNA targeting of cyclin D1 may depend upon the endogenous cyclin D1 expression levels.

## Discussion

TRUE gene silencing is one of the RNA-mediated gene expression control technologies. In the present study, among our designed sgRNAs cyclin D1, sgHT2 and sgHT5 were found to be effective inhibitors of cyclin D1 expression in SCC cells, suggesting that the binding sites of sgRNAs are important in efficiently guiding their action in the TRUE gene silencing method. The RNA interference (RNAi) method, using siRNA as a major tool, is also used to regulate gene expression. Although transfection of siRNAs is known to result in interferon (IFN)-mediated activation of the Jak–Stat pathway and global upregulation of IFN-stimulated genes [32], we have shown that 2′-O-methylated RNA does not induce interferon-β production in several cell types [24]. The TRUE gene silencing method using 2′-O-methylated sgRNA may be advantageous over a technology based on the inhibitory mechanism [24],[25]. Off-target effects are caused by nucleotide sequence similarity between the sgRNA molecule and short motifs in mRNAs of other genes not intended to be knocked-down. Because a sequence specificity of heptamer-type sgRNA is in theory assumed to be 12 bases and not 7 bases due to the demand of an upstream T-arm-like hairpin structure, there would be roughly one target site per a 4^12^ (∼1.7×10^7^) nucleotide sequence in the whole transcriptome with respect to each heptamer-type sgRNA [16]. Although this calculation implies that a sgRNA would target more than one mRNA species, considering the accessibility to target sites in folded mRNAs, off-target effects could be tolerable.

Cell cycle regulatory proteins are important candidates for therapeutic tumor suppressors. Deregulation of cyclin D1 has been observed to occur in a variety of cancer types including HNSCC [4]. Cyclin D1 activity is critical for tumor formation induced by other oncogenes (e.g. Ras), as mice deficient in cyclin D1 are resistant to tumorigenesis [33], [34]. In animal models, cyclin D1 has also been shown to exhibit oncogenic activity when overexpressed in specific tissues [35]. In this study, we demonstrated that well-designed sgRNAs targeting human cyclin D1 mRNA could down-regulate not only cyclin D1 expression but also CDK-mediated Rb phosphorylation. They also induced cell cycle G1-arrest, apoptosis and cellular proliferation in HNSCC cells. Many studies have indicated a significant association between high cyclin D1 expression and clinical outcome of patients not only in HSC but also several other types of cancers such as lung cancer, pancreatic cancer, breast cancer, melanoma and multiple myeloma. Our results indicate that sgRNAs targeting human cyclin D1 may have potential therapeutic application for various types of cancers.

Prior reports have demonstrated *in vitro* [36]–[41] and/or *ex vivo* [42]–[44] inhibition of cyclin D1 protein expression and decreased cell proliferation with antisense single-stranded oligonucleotides or siRNA for cyclin D1 mRNA in various cancer cells. A variety of transfection techniques has been used, including calcium phosphate co-precipitation, lipofection, retroviral and adenoviral infection [36]–[41], [45]. Although transfection agents are required to produce effects of antisense oligonucleotides or siRNA in most cultured cells, several investigations have shown that certain oligonucleotides are taken up and exert their effects without the need for any transfection agent [46]–[49]. Previously, we have also shown that a heptamer type of 2′-O-methylated sgRNA, mh1(Bcl-2), which targets human Bcl-2 mRNA, can be taken up by cells without any transfection reagents and that it can induce apoptosis of leukemia cells [24]. The results of our fluorescence imaging analysis show that 2′-O-methylated sgRNA can be taken up easily by cells without any carrier reagents. Several endocytosis inhibitors including chlorpromazine, nystatin or methyl-β-cyclodextrin were unable to diminish the apoptosis-inducing or target mRNA-reducing effects induced by naked sgRNA (Tamura et al., unpublished data), suggesting that functional uptake by cells in culture does not appear to be mediated by clathrin-, caveolae- or lipid raft-dependent endocytosis pathways. Similar to this, Koller *et al.* [50] also reported that functional uptake of a single-stranded phosphorothioate-modified antisense oligonucleotide is not mediated by clathrin- or caveolin-dependent endocytosis. Taken together, these data lead us to speculate that sgRNA might be taken up by SCC cells via a similar mechanism.

Following cellular uptake, sgRNAs are sequestered in intracellular compartments, from which they are thought to escape by a still elusive and presumably inefficient mechanism [51]. From our observation, sgRNAs were localized in vesicle-like structures outside the nucleus. It has been reported that phosphorothioate oligonucleotides are localized in acidic compartments, compatible with lysosomes [52]. Our previous studies also suggest that functional uptake of sgRNA is mediated by an intracellular vesicular transport process, since cellular responses induced by sgRNA are blocked by chloroquine and brefeldin A (Tamura et al., submitted, unpublished data). Therefore, intracellular compartments may mediate transport of sgRNA in SCC cells. A better understanding of how naked sgRNAs enter cells and how they reach their target RNA will aid in the design of more specifically-targeted and more potent sgRNA drugs for cancer therapy.

Cisplatin is one of the most potent antitumor agents known, displaying clinical activity against a wide variety of solid tumors. Its cytotoxic mode of action is mediated by its interaction with DNA to form DNA adducts, primarily intrastrand crosslink adducts, which activate several signal transduction pathways and culminate in the activation of apoptosis [53]. We examined whether effective sgRNAs work additively or synergistically with cisplatin. Although, in most cases, sgRNA with cisplatin showed only an additive effect, sgHT2 and sgHT5 worked synergistically in reducing cell viability of HSC-3 cells. This suggests that the combination of sgRNA and cisplatin may act cooperatively in inhibiting tumor cell proliferation. Therefore, combinations of anticancer drugs and sgRNA targeting cyclin D1 would be worth further investigation for their potential as therapeutic agents in SCC. Although sgRNAs based on TRUE gene silencing appear to be potential therapeutic agents, animal experiments to evaluate the systemic administration effect, toxicity, and pharmacokinetics are needed before clinical trials. Ultimately the best forms of therapeutic sgRNAs for SCC may be combined with other targeted drugs made of small molecules or antibodies.

## Supporting Information

Figure S1 **Confocal microscopic analysis for uptake and intracellular localization of sgRNA in normal cells.** Human fibroblastic cells were plated and cultured for 24 h. Naked Alexa568-3′-labeled sgH5 was added at 200 nM and then the cells were cultured for another 24 h, after which the cells were observed by confocal microscopy as described in Materials and Methods.
(TIF)

Methods S1 **Materials and Methods for Supporting Information.**
(RTF)

Video S1 **3-Dimensional analysis of intracellular localization of sgRNA.** HSC-3 cells were plated and cultured for 24 h. Naked Alexa568-3′-labeled sgH5 was added at 200 nM and then cultured for 24 h. The nuclei were stained with Hoechst33342 and, then observed by confocal microscopy and visualized by 3D imaging analysis. The sgRNA-Alexa568 and nuclei are shown in red and blue, respectively.
(WMV)

Video S2 **Dynamics of sgRNA localization in living cells (time-lapse analysis).** HSC-3 cells were plated and cultured for 24 h. Naked Alexa568-3′-labeled sgH5 was added at 200 nM and then cultured. Cells were observed by confocal microscopy and images were collected every 10 min from 6 to 24 h after sgRNA transfection. (Apoptosis was induced in HSC-3 cells at 10 sec into the movie).
(AVI)
