# Supplementary figures and images for: Growth Inhibition of Head and Neck Squamous Cell Carcinoma Cells by sgRNA Targeting the Cyclin D1 mRNA Based on TRUE Gene Silencing
Source: PLoS One. 2014 Dec 1;9(12):e114121. doi: 10.1371/journal.pone.0114121 (PMC4250192; doi:10.1371/journal.pone.0114121)

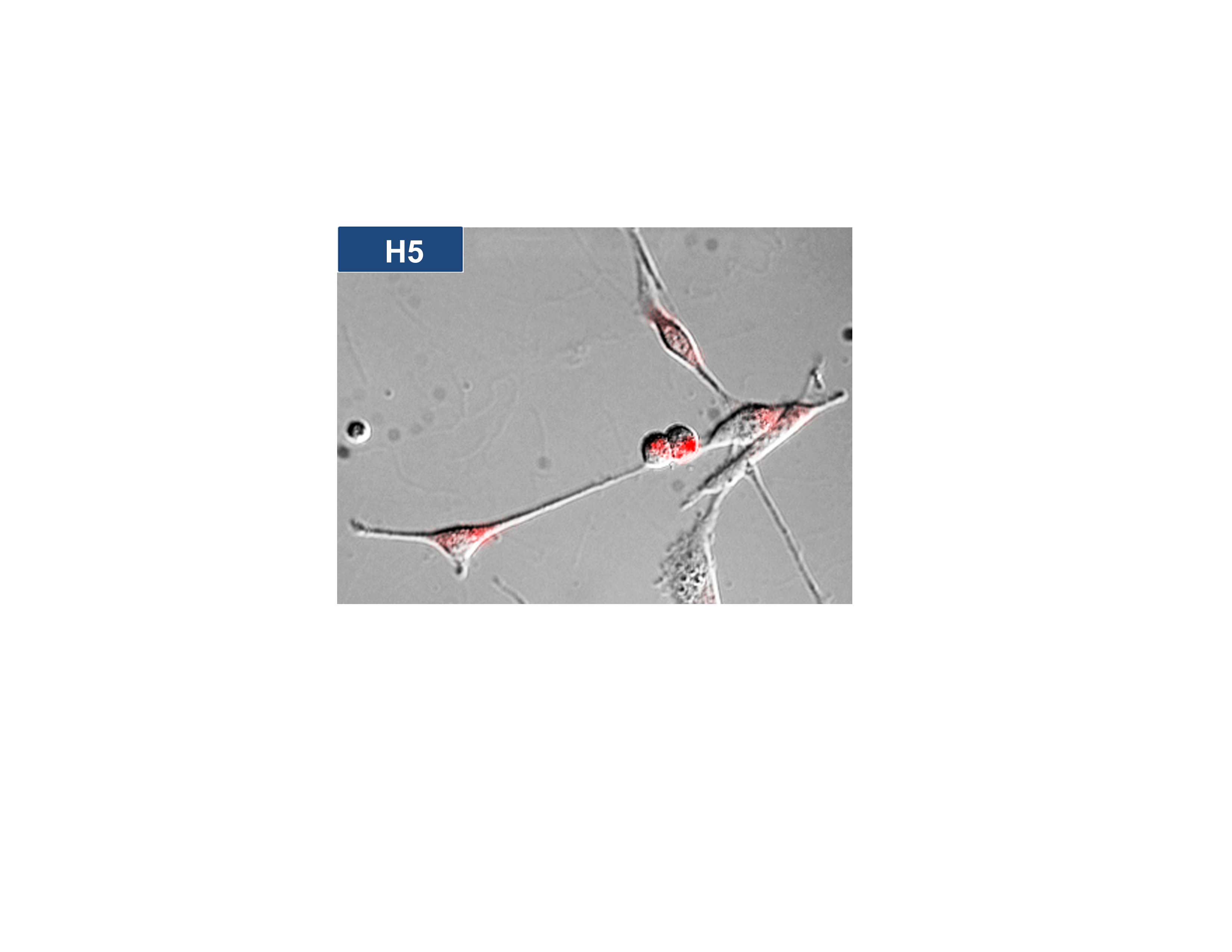

Supplement: Figure S1 — Confocal microscopic analysis for uptake and intracellular localization of sgRNA in normal cells. Human fibroblastic cells were plated and cultured for 24 h. Naked Alexa568-3′-labeled sgH5 was added at 200 nM and then the cells were cultured for another 24 h, after which the cells were observed by confocal microscopy as described in Materials and Methods. (TIF) [file pone.0114121.s001.tif]
