# Supplementary material for: Growth Inhibition of Head and Neck Squamous Cell Carcinoma Cells by sgRNA Targeting the Cyclin D1 mRNA Based on TRUE Gene Silencing
Source: PLoS One. 2014 Dec 1;9(12):e114121. doi: 10.1371/journal.pone.0114121 (PMC4250192; doi:10.1371/journal.pone.0114121)
Supplement: Methods S1 — Materials and Methods for Supporting Information. (RTF) [file pone.0114121.s002.rtf]

Methods S1
3-Dimensional analysis of sgRNA intracellular localization 
Confocal images were taken by a confocal laser scanning microscopy system (Nikon A1 and Ti-E, Nikon) equipped with an Apo TIRF x60 objective lens (NA 1.49, Nikon) and a stage-top incubator (INUBG2H-TIZB, Tokai Hit). Images were reconstructed in 3D by NIS-Elements ver. 4.11 (Nikon). 

Dynamics of sgRNA localization in living cells (time-lapse analysis)
HSC-3 cells (1 × 10^5^ cells/well) were seeded into collagen coated glass-bottomed dishes (Matsunami glass Inc.). After 24 h, the cells were treated with 200 nM naked Alexa568-3'-labeled sgRNA. The nuclei or mitochondria were visualized with Hoechst33342 or MitoTracker Green FM, respectively. The cells were rinsed twice with 1 × PBS, and an inverted microscope (Nikon A3, Ti-E) equipped with a Plan Fluor objective lens (NA 0.75, Nikon) and micro scanning stage (BI XY stage, Chuo Precision Industrial Co. Ltd.) was used to observe fluorescence images in living cells maintained at 37°C with a continuous supply of 95% air and 5% CO2 using a stage-top incubator (INUBG2TF-WSKM, Tokai Hit). Images were captured by a cooled charge-coupled device (CCD) camera (ORCA-R2, Hamamatsu Photonics). Images were collected every 10 min from 6 to 24 h after sgRNA treatment. The time-lapse images were acquired and arranged sequentially in a movie sequence on MetaMorph software (Molecular Devices, Sunnyvale, CA). The focus, contrast and brightness settings were kept constant during the course of image acquisition.
